# Single-cell transcriptomic landscapes of a rare human laryngeal chondrosarcoma

**DOI:** 10.1007/s00432-021-03883-1

**Published:** 2021-12-21

**Authors:** Chen Lin, Zhisen Shen, Yanguo Li, Shanshan Gu, Yaqin Lu, Hongxia Deng, Dong Ye, Qi Ding

**Affiliations:** 1 Lihuili Hospital of Ningbo University, Ningbo, China; 2 Institute of Drug Discovery Technology, Ningbo University, Ningbo, China; 3 The Ningbo Diagnostic Pathology Center, Ningbo, China

**Keywords:** Laryngeal chondrosarcoma, Single-cell sequencing and analysis, Immunohistochemistry, *SLAMF9* gene, Tumor microenvironment

## Abstract

**Propose:**

Laryngeal chondrosarcoma is a rare non-epithelial malignant tumor. At present, the cell type composition and molecular mechanism of laryngeal chondrosarcoma have not been systematically studied.

**Methods:**

This study focused on the histopathological and imaging features of a rare primary laryngeal chondrosarcoma in a 74-year-old male. The tumor and its paracancerous cartilage tissue were single-cell sequenced and analyzed and a total of 5455 single cells were obtained. Immunohistochemical levels were also verified.

**Results:**

In total five cell types were identified, including chondrocytes, myeloid cells, fibroblasts, lymphocytes, and endothelial cells. We carried out further subgroup analysis, focusing on the classification and differentiation of chondrocytes, functional enrichment analysis, and cellular communication analysis of all cell types, and explored the tumor microenvironment (TME) of laryngeal chondrosarcoma. Immunohistochemistry revealed the *SLAMF9* gene was specifically expressed in non-immune cells of chondrosarcoma, but was barely expressed in the normal cartilage tissues adjacent to chondrosarcomas.

**Conclusion:**

This single-cell sequencing approach provides clues for deciphering the potential mechanisms of tumor heterogeneity and TME composition in laryngeal chondrosarcoma, and represents an important step towards the treatment of laryngeal chondrosarcoma.

**Supplementary Information:**

The online version contains supplementary material available at 10.1007/s00432-021-03883-1.

## Introduction

Laryngeal chondrosarcoma is an extremely rare non-epithelial malignant tumor, accounting for 0.2% of laryngeal malignant tumors (Dubal et al. 2014). The disease occurs principally in elderly men. Hoarseness and dyspnea are the most common clinical symptoms. Other symptoms include dysphagia, neck lumps, and loss of consciousness. The course of the disease generally develops slowly with a favorable prognosis. Currently, surgical resection is the main treatment, while the 5-year survival rate can reach more than 90%, but its incidence is hidden and is not easily detected in the early stages, and its etiology is still unknown (Berge et al. 1998; Sanaat et al. 2009; Thompson and Gannon 2002). Therefore, exploring the transcriptional characteristics of the disease is of great value for understanding the mechanisms underlying tumorigenesis and the development of clinical treatments in the future.

Rapid development of single-cell sequencing technology provides a powerful tool for exploring genetic and functional heterogeneity, detecting rare cell subsets and reconstructing evolutionary lineages (Navin 2015; Tanay and Regev 2017). A recent study (Puram et al. 2017) on head and neck cancer (HNC) constructed a single-cell transcriptome map of HNC for the first time, revealing different types of cells in primary HNC and their metastatic tumors. However, such studies (Cillo et al. 2020; Puram et al. 2017) reporting HNC single-cell transcriptology analyses are of epithelial origin, and cartilage-derived laryngeal chondrosarcoma has not been explored.

Herein, we studied of the tumor tissue and paracancerous tissue of a 74-year-old man with primary laryngeal chondrosarcoma, and discussed its histopathological and imaging features. A total of 5455 single cells were obtained and 5 cell types corresponding to 17 clusters were identified by single-cell sequencing of the tissues in this case of laryngeal chondrosarcoma. It includes chondrocytes, myeloid cells, fibroblasts, lymphocytes, and endothelial cells. We were the first to carry out the cell subgroup cluster analysis of laryngeal chondrosarcoma, focusing on the classification and differentiation of chondrocytes, exploring the changes of TME of laryngeal chondrosarcoma, and enriching the function of myeloid cells, chondrocytes and lymphocytes. Secondly, through the analysis of cell communication, we found that myeloid cells are the cell type which communicate most closely with chondrocytes, and separately explored the receptor pairs of myeloid cells for signal transduction to chondrocytes, to predict target genes corresponding to ligands. In short, this single-cell sequencing analysis provides clues for deciphering the potential mechanism of intra-tumor heterogeneity and TME composition in laryngeal chondrosarcoma, and provides valuable resources for the treatment of laryngeal chondrosarcoma.

## Materials and methods

### Specimen collection

In this study, volunteer patients signed consent forms at the Li Huili Hospital of Ningbo Medical Center, and the study was approved by the hospital ethics committee (NO.NBU-2020-059). Informed consent was obtained for the 74-year-old patient. Following laryngeal tumor resection, the surgeon collected tumor tissue and paracancerous tissue samples, to confirm the histopathology and diagnosis. All freshly resected biopsies were used for single-cell sequencing, immunohistochemistry, and routine pathology.

### Single-cell suspension preparation, cell sorting, reverse transcription, and amplification

Fresh tissue specimens were cut into pieces, washed in DPBS 2-3 times, stored in 4℃ preservation solution, and transported to the laboratory on ice. In accordance with the instructions of Meitianmei Human tumor dissociation Kit (MiltenyiBiotec, Cat.no.130-095929), each tissue was cut into about 2–4 mm pieces then digested and dissociated in a digestive solution containing RPMI1640, EnzymeH, EnzymeR, EnzymeA at 37 ℃ for 30 min. Tissue digestion, preparation of single-cell suspension, cell sorting, reverse transcription, and amplification were carried out.

### Single-cell RNA-seq data processing

The 10 × Genomics analysis software CellRanger (version3.0.2) was used to process the original data, and the FASTQs data from Illumina sequencing were compared, processed, and initially integrated with the human reference genome. Next, the data processed by CellRanger were reorganized using the Seurat (version3.1.1) package via RStudio (version1.4.1106) for further dimensionality reduction, clustering, and analysis. We used the scDblFinder package to filter double-cell data, and eliminate the interference of ribosomes, mitochondria, blood cells, and other genes in the process of quality control. The standards we set are as follows: all genes expressed in less than one cell were removed, and the number of genes expressed in each cell was set between 500 and 5000. The unique molecular identifier (UMI) count was less than 500 and the proportion of mitochondrial gene expression in single cell was set at about 15%.

### Nonlinear dimensionality reduction (t-SNE/UMAP) recognition of cell types and subtypes

In order to visualize the data, we used Seurat software to further reduce the dimension of 5455 cells, and SCTransform was used to calculate the gene expression value. The dimension was reduced by Harmony principal component analysis. Harmony algorithm uses principal component analysis to embed transcriptome expression spectrum into low-dimensional space, and then removes the unique influence of data set via iterative process. After reducing the variables, the homogenized expression value was used for principal component analysis (PCA). The first 10 principal components were selected from the results of PCA analysis for subsequent clustering and analysis. We mainly use two dimensionality reduction algorithms, t-SNE and UMAP, and identify cell clusters through the clustering algorithm optimized based on the shared nearest neighbor (SNN) module. Finally, we obtained 17 cell clusters. Subsequently, all the cell subsets were re-clustered using the same approach. We preliminary identified cell types/subtypes by combining SingleR package and in the reference of CellMarker database, then further classified them by combining functional analysis. In the identification of chondrocyte subsets, we also referred to the cell markers provided by Ji (Ji et al. 2019) for auxiliary identification.

### Functional annotation and pathway analysis

Gene Ontology (GO) functional annotation is based on the GO database, and included biological processes, cellular components, and molecular functions. The Fisher exact test was used to select important categories, and GO terms, with *Q* values < 0.05 were considered important. The Kyoto Encyclopedia of Genes and Genomes (KEGG) is a database resource for understanding the advanced functions and functions of biological systems (http://www.genome.jp/kegg/). Pathways with a *Q* value ≤ 0.05 were considered significantly enriched. Both tools are implemented by R language.

### Pseudotime analysis

Pseudotime analysis, was predicted by constructing the track of changes between cells. There are pseudo-temporal variations in the cells themselves, so we used R-packet monocle3 to analyze our subsets of cells, and then obtained the evolution trajectory of the cells.

### Cell communication

Cell–cell communication mediated by ligand-receptor complexes play a key role in biological processes, such as tumorigenesis, and surrounding inflammation. We compared differences in ligand and receptor gene expression and unlocked the crosstalk between cell types using the iTALK package. In addition, we further analyzed the communication between two specific subgroups of interest through the NicheNet package, which predicts ligand-receptor interactions between interacting cells by combining cell expression data with known signals and gene regulatory networks. Applying NicheNet to the number of tumor cells and to the immune cell microenvironment, we can infer the active ligand and its gene regulation on interacting cells.

### Immunohistochemical analysis

We used the EnVision two-step method for immunohistochemical staining of the paraffin-fixed samples. Samples were prepared by incubating tissue slices in 100℃ EDTA antigen repair fluid for 20 min and then allowing them to cool down under the room temperature. The SLAMF9 polyclonal antibody was purchased from PYRAM Company (Shanghai, China) at 1:100 working concentration. The second antibody, DAB + chromogen and its substrate buffer were all purchased from Dako (Glostrup, Denmark). The results of immunohistochemical staining were evaluated by 2 pathologists using a double-blind method.

## Results

### Histology, imaging, and single-cell sequencing features of human laryngeal chondrosarcoma

**Fig. 1:**
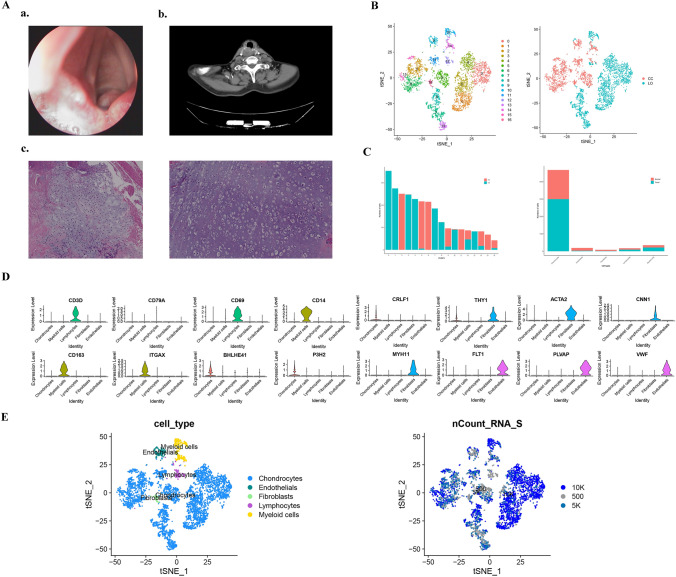
Single-cell RNA-Seq reveals the constituent cell types in laryngeal chondrosarcoma. **A** Histological and imaging features of human laryngeal chondrosarcoma. **a** The laryngoscope showed a mass protruding to the laryngeal cavity under the glottis; **b** the CT reconstruction pattern showed a soft tissue mass in the right subglottic wall, and the boundary was not clear; **c** Hematoxylin–eosin staining of the surgical specimens on the left revealed the cartilage tissue of the tumor, while the right side was the uninvolved adjacent normal cartilage tissue; **B** **a** A total of 17 cell clusters were identified; **b** t-SNE maps showed laryngeal chondrosarcoma tumor and paracancerous sample; *CC* chondrosarcoma, *LO* laryngeal chondrosarcoma; **C** The number of each cluster and 5 cell types in tumor and paracancerous samples; **D** The violin graph showing the expression of representative markers in each cell types. *X*-axis represents different cell types; *Y*-axis represents gene expression level; **E** **a** t-SNE diagram shows the main cell types in laryngeal chondrosarcoma; **b** Gene expression activity distribution map of the identified cells

The patient reported no history of smoking or alcohol consumption. Three months before the operation, laryngeal cricoid cartilage resection was performed under general anesthesia for laryngeal tumors. During the operation, several cartilage-like masses with clear round boundaries in the tracheoesophageal and retroannular area, and cartilage tumors were studied intraoperatively by frozen sections. For patients with laryngeal chondrosarcoma confirmed by pathology, alpha-fetoprotein (AFP) 13.1 μg/L, ferritin 643.0 μg/L, free prostate specific antigen (FPSA) 0.457 μg/L were measured. There was no deformity in the appearance of the larynx. Indirect laryngoscope showed no redness or swelling in the epiglottis, tumor protruding to the laryngeal cavity under the glottis, and a smooth surface (Fig. 1A_a). Computed tomography revealed a soft tissue mass in the right subglottic wall, the boundary was unclear, the adjacent tissue structure was pressed and squeezed, and inhomogeneous enhanced changes of the lesions could be seen on contrast-enhanced scan (Fig. 1A_b). The size of the tumor was 2.0 × 1.8 × 1.1 cm, including bone tissue. Cellular atypia of chondrocytes was obvious in the lesion to the adjacent normal cells (Fig. 1A_c). The patient described herein is currently alive, suggesting a diagnosis of an inert malignant tumor.

After the initial quality control of the overall characteristics of laryngeal chondrosarcoma and adjacent cartilage tissue, we obtained a total of 5455 cell single-cell transcripts from the laryngeal chondrosarcoma and normal tissue sample, and a total of 24,080 genes were detected. We used Seurat software to analyze this single-cell data after data pre-processing. Finally, we identified 17 major cell clusters after principal component analysis (Fig. 1B). Based on the typical cell markers listed (Table 1), a total of 2022 cells in clusters 1–8, 11, 13 and 15 were identified as chondrocytes, and there were, respectively 2981 and 1673 cells in tumor and tumor samples. In addition, 350 cells in clusters 10 and 14 were classified as myeloid cells, accounting for 6.42% of the total cells (Table 2). There were also fibroblasts (85, 1.56%), endothelial cells (192, 3.52%), and lymphocytes (174, 3.19%) (Fig. 1C, D). Compared to normal samples, tumor samples contained a higher proportion of chondrocytes and myeloid cells, while other types of cells were more frequent in adjacent normal tissue. The types and number of cells are shown in Table 2.

**Table 1:** Known markers for cell population identification

Cell Type	Clusters	Marker Genes	References
Chondrocytes	1–8,11,13,15	COMP, P3H2, BHLHE41, CRLF1	Posey et al. (2018), Sato et al. (2016), van Hoolwerff et al. (2020)
Fibroblasts	16	ACTA2, CNN1, MYH11	Korosec et al. (2019), Nurmik et al. (2020)
Endothelial cells	9	FLT1, PLVAP, VWF	Kivela et al. (2019), Songstad et al. (2015)
Myeloid cells	10,14	CD14, CD163, ITGAX	Nielsen et al. (2020), Zhang et al. (2019)
Lymphocytes	12	CD3D, CD79A, CD69	da Silva et al. (2020)

**Table 2:** Cell clusters distribution in laryngeal chondrosarcoma and normal tissue

Cell type	Adjacent	Adjacent ratio (%)		Tumor	Tumor ratio (%)
Chondrocytes	1673	30.67	2981		54.65
Endothelials	163	2.99	29		0.53
Fibroblasts	70	1.28	15		0.27
Myeloid cells	135	2.47	215		3.94
Lymphocytes	81	1.48	93		1.70

### Aggregation and state analysis of chondrocytes

**Fig. 2:**
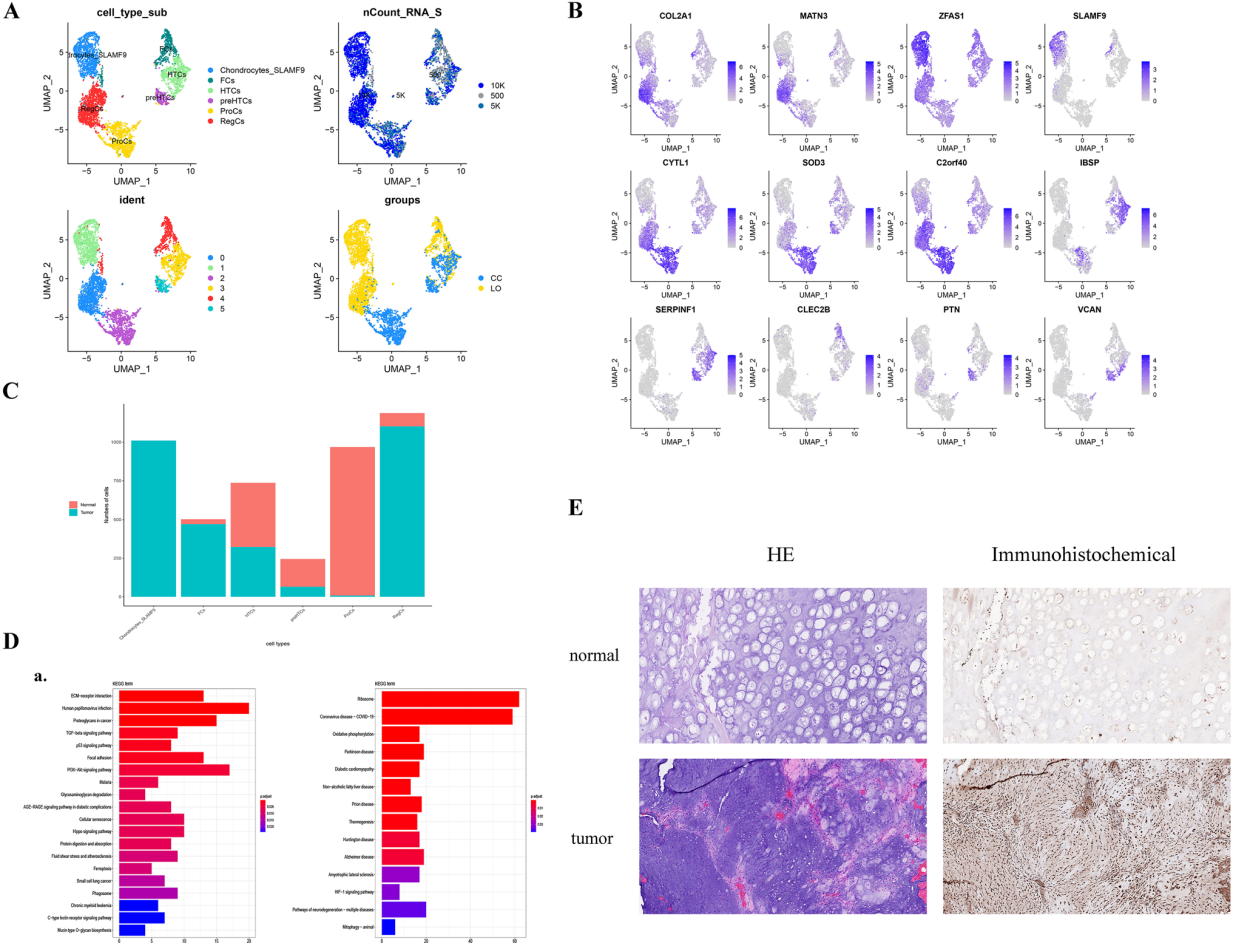
Chondrocyte clustering and status in laryngeal chondrosarcoma. **A** t-SNE diagram showing the clusters and main subgroups of chondrocytes in laryngeal chondrosarcoma and their corresponding tissue sites; **B** UMAP showing the expression of representative markers in the cell subtypes of chondrocytes identified by laryngeal chondrosarcoma; **C** Histogram showing the main subsets of chondrocytes in laryngeal chondrosarcoma and the content of each subgroup in chondrosarcoma tissue and adjacent tissue; **D** KEGG pathway analysis of RegCs and Chondrocytes_SLAMF9 in laryngeal chondrosarcoma; **E** Immunohistochemical staining of SLAMF9 in chondrosarcoma and adjacent tissues

Chondrocytes were the most common cell type in this single-cell identification, with a total of 4654 chondrocytes detected, accounting for the largest proportion of cell types in both tumor and paracancerous cartilage (Table 2). Clustering revealed 6 subclusters, which also indicated the functional heterogeneity of the chondrocyte population (Fig. 2A). Further investigation of the expression and distribution of chondrocyte markers in six clusters (Fig. 2B), which when combined with the function of each chondrocyte cluster, they can be divided into regulatory chondrocytes (RegCs), SLAMF9 specific chondrocytes (Chondrocytes_SLAMF9), proliferative chondrocytes (ProCs), hypertrophic chondrocytes (HTCs), fibrocartilage chondrocytes (FCs), and prehypertrophic chondrocytes (preHTCs).

As shown in Fig. 2C, the content of the prehypertrophic, hypertrophic, and proliferative chondrocytes in normal tissue was higher than that in tumor tissue, specifically, mainly ProCs existed in normal tissue. To describe its function in more detail, we evaluated phenotypic diversity among subpopulations through functional enrichment. We found that the differentially expressed genes of ProCs were concentrated in functions associated with the detoxification of inorganic compounds, reaction and stabilization of cells to various metal ions, and the metabolic processes of reactive oxygen species. Further, protein processing in the endoplasmic reticulum, mineral absorption and ferroptosis processes were enriched in the pathway, which suggested that this type of chondrocyte existed in the normal tissue adjacent to the cancer, and maintained the normal living environment of the surrounding cells by detoxifying and stabilizing the activity of metal ions, and may achieve anti-tumor effects by inducing iron death. Furthermore, preHTCs are the precursors of HTCs, and these two kinds of cell subtypes often appear in degenerative joint diseases (Neumann et al. 2015). When chondrocytes are stimulated by specific biochemical stimulation, the local microenvironment, or other external stimulation, it will enter the stage of proliferation and hypertrophy. Finally, hypertrophic chondrocytes undergo apoptosis, necrosis, cartilage degradation, and further calcification. These two subtypes of chondrocytes indicate a tendency for tissue calcification.

Accordingly, the contents of RegCs, Chondrocytes_SLAMF9 and FCs in tumor tissue were significantly higher than those in normal tissue, among which Chondrocytes_SLAMF9 were only present in tumors. Pseudotime analysis (Fig. S2C) showed that these two chondrocyte types were basically located in the initial stage of differentiation, which suggested that tumor chondrocytes in laryngeal chondrosarcoma may be dedifferentiated. Dedifferentiation is exactly the theoretical basis for tumorigenesis (Liu 2018). Additionally, RegCs are enriched in Extracellular matrix (ECM)-receptor interactions, Human papilloma virus (HPV) infection, proteoglycans in cancer, the TGF-beta signaling pathway, the p53 signaling pathway, and other signaling pathways (Fig. 2D_a). It suggests that RegCs in tumor may induce tumor progression by promoting cell adhesion and the secretion of proteoglycan between tumor cells, but also indicates RegCs adjacent to cancer tissues may activate TGF-beta, p53, and other pathways to promote apoptosis and inhibit tumor development. Herein, we also found that HPV infection was not only a factor affecting head and neck squamous cell carcinomas, but was also associated with laryngeal chondrosarcoma. In addition, pathway analysis (Fig. 2D_b) showed that there was significant enrichment in ribosome, Coronavirus disease-COVID-19, and oxidative phosphorylation pathways in Chondrocytes_SLAMF9. *SLAMF9* is specifically and highly expressed in the chondrocyte subsets of tumor samples. In previous studies, *SLAMF9* was reported to mediate immune response by regulating homeostasis of plasmacytoid dendritic cells (pDC) (Sever et al. 2019), regulating common macrophages (Zeng et al. 2020) and tumor-associated macrophages (Dollt et al. 2018). A review of signaling lymphocytic activation molecule (SLAM) family proteins by Dragovich and Mor (2018) suggested that *SLAMF9* was a potential therapeutic target for inflammatory and autoimmune diseases. We speculate that in whole laryngeal chondrosarcoma, in addition to macrophages, lymphocytes, and other immune cells involved in the immune response or in immune escape, Chondrocytes_SLAMF9 subsets are a participant in the tumor immune microenvironment, and shows certain potential in tumor immunotherapy.

We detected the expression of SLAMF9 protein in 20 chondrosarcoma tissues by immunohistochemical staining (Fig. 2E). We found that SLAMF9 was positive in all chondrosarcomas evaluated, and showed a specific distribution in chondrosarcoma chondrocytes, but minimal expression in normal cartilage tissue adjacent to cancer. We speculate that SLAMF9 is a specific marker in non-immune cells of chondrosarcoma and shows great potential for interfering with tumor function.

### Tumor microenvironment and clinical significance of laryngeal chondrosarcoma

In this single-cell identification study, the percentage of myeloid cells, lymphocytes, endothelial cells, and fibroblasts were respectively 6.41%, 3.18%, 3.52%, and 1.55%. The crosstalk between these cells and chondrocytes constituted the TME of laryngeal chondrosarcoma. The number of fibroblasts was small, and fibroblasts functions were mainly involved in muscle contraction, muscle system development, promoting platelet aggregation, and intercellular adhesion (Fig. S1A). Besides, endothelial cells are vascular endothelial cells, which participate in the composition of the ECM, endothelial development, regulation of vascular development, and other processes (Fig. S2B). The distribution in the adjacent normal tissue is significantly greater than that of tumor tissue (Fig. S2C), which may be related to tumor angiogenesis often occurring at the edge of the tumor. Further subgroup analysis subdivides lymphocytes into B cells, T cells, and NK cells, and there are significant differences in genes expression across subsets (Fig. S1D).

**Fig. 3:**
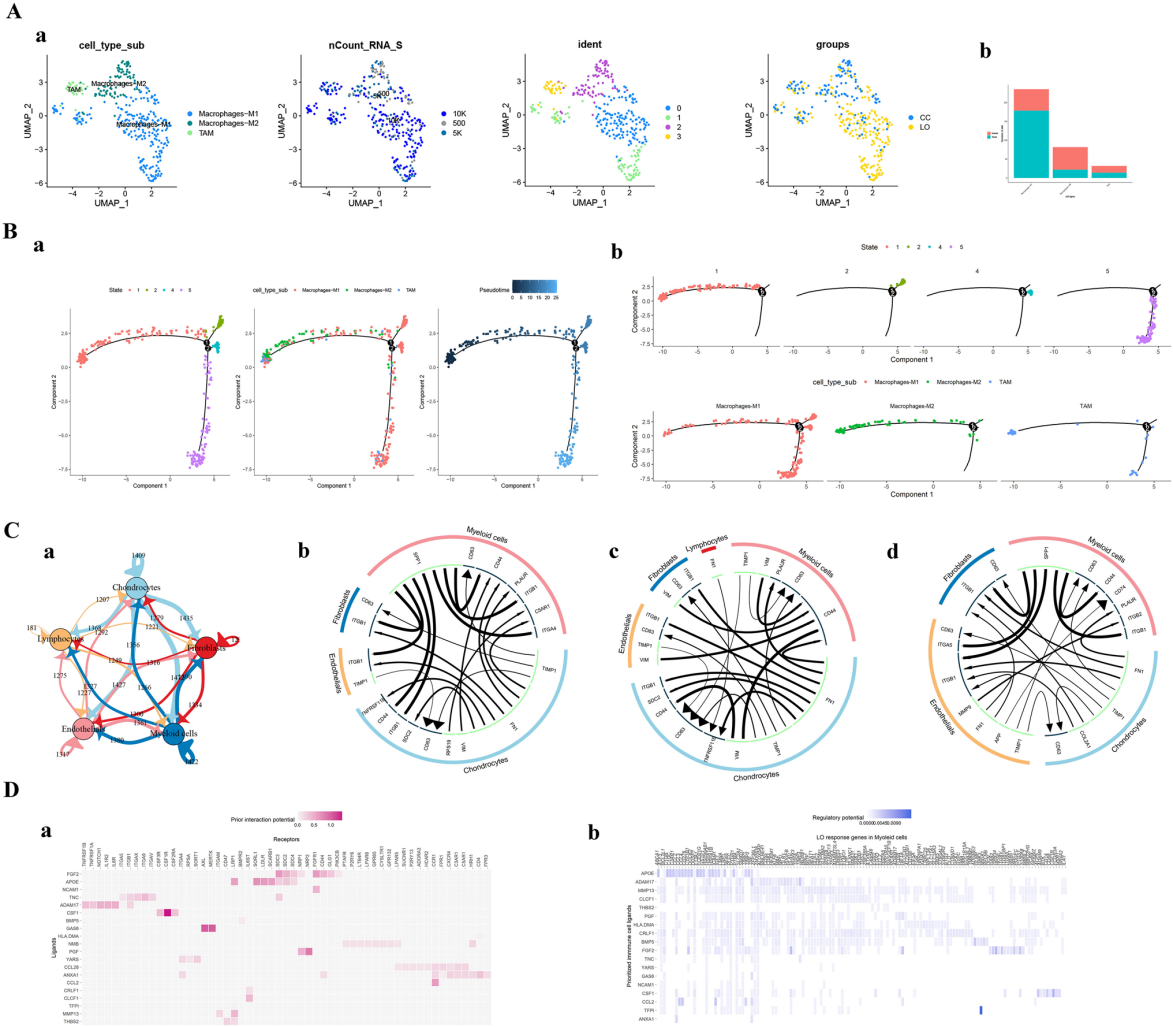
Functional analysis and clinical significance in chondrocytes and cancer cells. **A** **a** UMAP cluster diagram of subsets of myeloid cells; b. Proportion of myeloid cells subsets in tumor and paracancerous tissue; **B** pseudotime analysis trajectory map showing the differentiation status of myeloid cells; **C** **a** overview of cell communication; **b** Top ligand-receptor interactions in tumor and paracancerous areas; c. Top ligand-receptor interactions in laryngeal chondrosarcoma; **d** top ligand-receptor interactions in paracancerous tissue; **D** **a** Predictive heatmap of ligand-receptor pairs among myeloid cells and chondrocyte subsets; **b** Prediction heatmap of target genes targeting chondrocytes with myeloid cell ligands

Myeloid cells are the most numerous cell types and rank only second to chondrocytes. Myeloid cells are mainly identified as M1 macrophages, M2 macrophages, and tumor-associated macrophages (TAM). Based on the pseudotime analysis (Fig. 3B), the differentiation stages of M1 macrophages were more diverse. Most TAMs were immature, while a small number may have mutated from well-differentiated M1 macrophages. Subsequently, we analyzed intercellular communication and found that myeloid cells were the cell type with the most interactions with chondrocyte ligand receptors. *SPP1*, a TAM-specific protein, communicates significantly with *CD44* and *ITGB1* in chondrocytes (Fig. 3C). *CD44* is related to tumor heterogeneous adhesion and other functions, while *ITGB1* has been confirmed to be involved in the occurrence and development of gastric, pancreatic, and bladder cancers, and is closely related to cellular immunity and immunosuppression (Guo et al. 2020; Zhuang et al. 2020). In addition, we conducted a further analysis of the communication relationship between myeloid cells and chondrocytes, and obtained a prediction of the protein–protein interaction (PPI) network (Fig. 3D_a) supported by the ligand pairs described in the literature. In addition, the corresponding target genes (Fig. 3D_b) that may be regulated by ligands were identified, in which the predictive score of CCL2 targeting GRK3 was highly significant. More experiments are needed to verify the mechanism of macrophages in laryngeal chondrosarcoma. As the case is very rare, we could not pursue such experiments in our study.

## Discussion

Tumor heterogeneity is not only a research hotspot and a complex property of malignant tumors, but it is also a major challenge in tumor diagnosis and treatment. As an emerging technology, single-cell sequencing technology has unique advantages in analyzing tumor cell subsets and studying tumor occurrence, evolution, and drug resistance, and provides new ideas for clinical diagnosis, treatment and prognosis of tumors. As a rare non-epithelial malignant tumor, laryngeal chondrosarcoma is characterized by hoarseness and dyspnea. Total laryngectomy is recommended for more than half of annular lesions and is the preferred treatment over 29.4% portion of patients. For Galletti et al., multidisciplinary evaluation by chemoradiologist and oncologist is preferable to evaluate new surgical or chemoradiotherapy treatments in some cases (Galletti et al. 2019). Currently, few studies have investigated its molecular characteristics, and there is a lack of consensus on study findings as data refer to a small number of case reports (Arslan et al. 2008; Madrigal et al. 2002; Maroun et al. 2019).

In this study, we investigated tumor and paracancerous tissue of laryngeal chondrosarcoma by single-cell transcriptome sequencing. The cell landscape of human laryngeal chondrosarcoma was described at the single cell level for the first time, and 5 cell types were identified, including chondrocytes, lymphocytes, myeloid cells, fibroblasts, and endothelial cells. Chondrocytes are the most common cell type, and subpopulation re-clustering indicates the presence of a small number of other cell types and functional cells in cartilage tissue, which suggests significant diversity in the tumor immune microenvironment. The chondrocyte cluster with specific expression of the immune gene *SLAMF9* attracted our attention, and we found that the protein was specifically expressed in chondrosarcoma tissues by immunohistochemical staining. Secondly, our functional enrichment and pathways analysis of the chondrocytes and other cells revealed a certain heterogeneity in each subgroup of chondrocytes. Each subgroup may not only promote or inhibit the occurrence and development of tumor through different functions and pathways, but each may also participate in the immune response. Abnormal ossification of laryngeal cartilage is considered to be a possible cause of tumorigenesis (Potochny and Huber 2014). Histopathological analysis revealed that as the number of chondrocytes increased, more abnormal cells were detected, nuclear chromosomes condensed, and pathological mitosis appeared. Hypertrophic chondrocytes are generally regarded as the terminal state before cartilage ossification, and the identification of this cell type also supports the above hypothesis to a certain extent. Through the analysis of cell communication, we determined that myeloid cells were the most significant cell type that interacted with the ligand receptors of chondrocytes. We used NicheNet to predict target genes regulated by myeloid-derived ligands and found that the predictive score of *GRK3* regulated by *CCL2* was very significant. GRK3 is a type of G-protein-coupled receptor kinase, which is associated with rapid desensitization of G-protein-coupled receptors. In cancer, GRK3 can promote the proliferation and growth of colon cancer cells) Jiang et al. 2017) and influences the metastasis of triple negative breast cancers (Billard et al. 2016), although, it is also a tumor suppressor gene in hepatocellular carcinoma (Jin et al. 2017). Further study on the role of *SLAMF9* and *GRK3* in the microenvironment and tumor progression of laryngeal chondrosarcoma may provide clues for laryngeal chondrosarcoma pathogenesis and potential new therapeutic targets.

In conclusion, this single-cell analysis revealed a rare transcriptome map of laryngeal chondrosarcoma. This map provides clues for further elucidating the mechanism of tumorigenesis and discovering biomarkers and therapeutic targets, and provides a better understanding of laryngeal chondrosarcoma.

## Supplementary Information

Below is the link to the electronic supplementary material.

Supplementary file1 (PDF 571 kb)

## Abbreviation

Chondrocytes_SLAMF9: SLAMF9 specific chondrocytes
