# Supplementary material for: Single-cell transcriptomic landscapes of a rare human laryngeal chondrosarcoma
Source: J Cancer Res Clin Oncol. 2021 Dec 21;148(4):783–92. doi: 10.1007/s00432-021-03883-1 (PMC8688141; doi:10.1007/s00432-021-03883-1)
Supplement: Supplementary file 1 — Supplementary file1 (PDF 571 kb) [file 432_2021_3883_MOESM1_ESM.pdf]

Supplementary Files

Cell Type	Clusters	Marker Genes
RegCs	0	COL2A1, MATN3
Chondrocytes_SMALF9	1	ZFAS1, SLAMF9
ProCs	2	CYTL1, SOD3
HTCs	3	C2orf40, IBSP
FCs	4	SERPINF1, CLEC2B
preHTCs	5	PTN, VCAN

Table S1. Markers of chondrocytes lineages

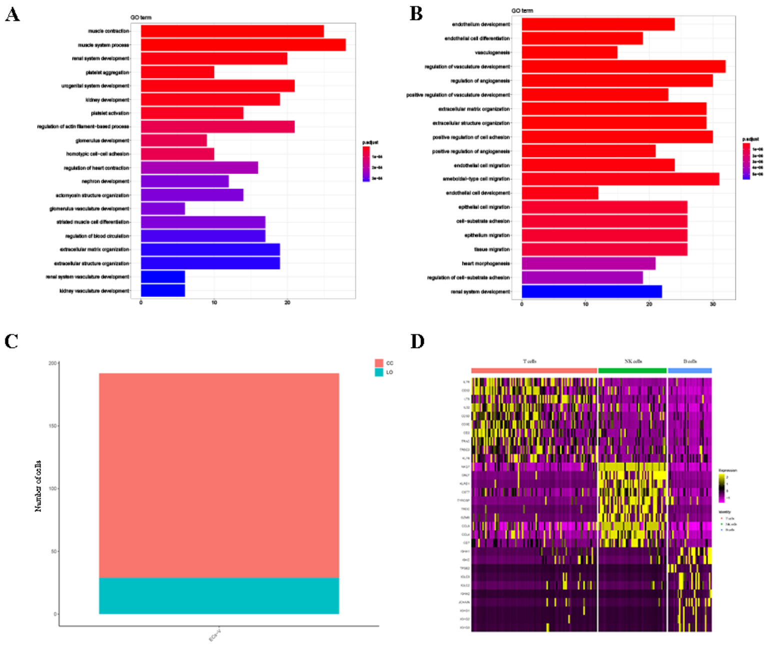

Fig.S1 Partial non-chondrocyte function in tumor microenvironment A.Gene Ontology enrichment of fibroblasts; B. Gene Ontology enrichment of endothelial cells; C. Distribution of endothelial cells in tumor and paracancerous tissue; ECs-V, Vascular endothelial cells; D. Heatmap of markable genes in Lymphocytes subtypes

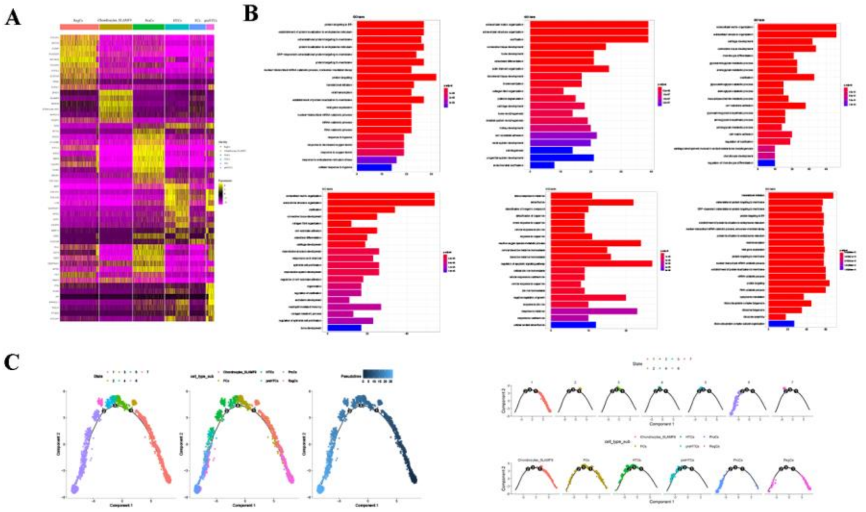

Fig.S2 Chondrocyte subtype functions and timing analyse A.Heatmap of top 10 differentiate genes of Chondrocyte subtypes; B.Gene Ontology enrichment of FCs, HTCs,

RegCs, preHTCs, ProCs, Chondrocytes_SLAMF9; C. Pseudotime analysis trajectory map showing the differentiation status of Chondrocytes
